# Pretreatment serum interleukin‐1*β*, interleukin‐6, and tumor necrosis factor‐*α* levels predict the progression of colorectal cancer

**DOI:** 10.1002/cam4.602

**Published:** 2016-01-22

**Authors:** Pei‐Hung Chang, Yi‐Ping Pan, Chung‐Wei Fan, Wen‐Ko Tseng, Jen‐Seng Huang, Tsung‐Han Wu, Wen‐Chi Chou, Cheng‐Hsu Wang, Kun‐Yun Yeh

**Affiliations:** ^1^ Division of Hemato‐oncologyDepartment of Internal MedicineChang Gung Memorial HospitalKeelung and Chang Gung UniversityCollege of MedicineKeelungTaiwan; ^2^ Department of NutritionChang Gung Memorial HospitalKeelung and Chang Gung UniversityCollege of MedicineKeelungTaiwan; ^3^ Division of Colorectal SurgeryChang Gung Memorial HospitalKeelung and Chang Gung UniversityCollege of MedicineKeelungTaiwan

**Keywords:** Colorectal cancer, CRP, IL‐1*β*, IL‐6, Progression, TNF*α*

## Abstract

The correlations of pretreatment serum concentrations of proinflammatory cytokines such as interleukin (IL)‐1*β*, IL‐6, and tumor necrosis factor‐*α* (TNF*α*) with the clinicopathologic features and progression of colorectal cancer (CRC) were investigated. The pretreatment serum levels of IL‐1*β*, IL‐6, and TNF*α* were measured in 164 CRC patients before treatment. The relationships between changes in proinflammatory cytokine and C‐reactive protein (CRP) levels and both clinicopathologic variables and disease progression were examined by univariate and multivariate analysis. Advanced tumor stage was associated with a poorer histologic differentiation, higher CRP level, lower albumin level, and inferior progression‐free survival rate (PFSR). Furthermore, high levels of CRP (>5 mg/L) were associated with proinflammatory cytokine intensity, defined according to the number of proinflammatory cytokines with levels above the median level (IL‐1*β* ≥10 pg/mL; IL‐6 ≥ 10 pg/mL; and TNF*α* ≥55 pg/mL). Under different inflammation states, proinflammatory cytokine intensity, in addition to tumor stage, independently predicted PFSR in patients with CRP <5 mg/L, whereas tumor stage was the only independent predictor of PFSR in patients with CRP ≥5 mg/L. Proinflammatory cytokine intensity and the CRP level are clinically relevant for CRC progression. Measurement of IL‐1*β*, IL‐6, and TNF*α* serum levels may help identify early cancer progression among patients with CRP <5 mg/L in routine practice.

## Introduction

Systemic inflammation is strongly linked to cancer development 1. Inflammatory mediators such as cytokines, reactive oxygen, and nitrogen species produced by tumor and immune cells of tumor stroma create a carcinogenic microenvironment that contributes to cancer initiation and progression. Genetic variation associated with this ongoing inflammatory process alters posttranslational mechanisms of DNA repair, inactivates tumor suppressor genes, and disturbs the apoptotic pathway 1, 2, 3. Colorectal cancer (CRC) exemplifies this process as chronic inflammatory bowel disease (IBD), classified as Crohn's disease and ulcerative colitis, increases the risk of CRC development, whereas nonsteroid anti‐inflammatory drugs inhibit CRC formation 1, 4. C‐reactive protein (CRP), an acute‐phase reactive protein produced by hepatocytes, is broadly used for diagnosing inflammation and cancers 5. It is upregulated in response to proinflammatory cytokines such as interleukin (IL)‐1*β*, IL‐6, and tumor necrosis factor‐*α* (TNF*α*) in CRC 5, 6. In addition, an increased serum CRP level is associated with advanced pathologic stage, more local tumor invasion, and a higher rate of recurrence rate of CRC patients 7, 8. Furthermore, a IL‐6 promoter polymorphism is correlated with serum IL‐6 levels, which may elevate the basal level of inflammation and increase the risk of CRC 9, 10. Finally, accumulated evidence demonstrates that elevated IL‐1*β*, IL‐6, and TNF*α* levels are associated with either tumor stage, survival rate, or distant metastasis in CRC patients 1, 11, 12.

Cancer patients with the same tumor stage may show varying responses and survival, despite the use of standard treatment in this pathologically homogenous population 1, 13. The search for novel tumor markers to identify cancer patients who are in need of additional medical attention following standard treatment at an early stage has continued to develop during the past decades, with an emphasis on various biological parameters, including the regulation between tumor promoter and tumor suppressor genes as well as the inflammation status and cytokine expression 1, 8. The aim of this study was to measure the pretreatment serum levels of IL‐1*β*, IL‐6, TNF*α* in CRC patients and examine the relationship between these proinflammatory cytokines and the clinicopathologic features of CRC, especially progression‐free survival.

## Materials and Methods

### Study participants

Data were retrospectively collected from 164 patients with CRC who visited Chang Gung Memorial Hospital in Keelung, Taiwan between January 2007 and December 2009. All patients were followed up until June 2014. The following parameters were recorded and analyzed for all study patients: age, sex, tumor location, tumor‐node metastasis (TNM) stage, histologic differentiation, white blood cell (WBC) count, albumin level, C‐reactive protein (CRP) level, carcinoembryonic antigen (CEA) level, TNF*α* level, IL‐1*β* level, IL‐6 level, and progression‐free survival (PFS). Progression‐free survival was defined as no imaging or pathological evidence of disease progression 3 years after diagnosis. Tumors were classified retrospectively according to the 7th edition American Joint Committee on Cancer Staging System based on findings of physical examination, routine laboratory tests, chest radiography, and computed tomography of the abdomen. The pathological diagnoses of all enrolled patients were reviewed and confirmed by the CRC committee at our institute. The committee members included two colorectal surgeons, three medical oncologists, two radiation oncologists, and two pathologists. This study was approved by the Institutional Review Board at Chang Gung Memorial Hospital in Taiwan.

### Measurements of pretreatment serum IL‐1*β*, IL‐6, and TNF*α* levels by enzyme‐linked immunosorbent assay (ELISA)

Blood was collected from patients before treatment and centrifuged at 500g for 15 min. All serum samples were stored at −80°C in pyrogen‐free plastic tubes until analysis. Serum IL‐1*β*, IL‐6, and TNF*α* levels were determined by DuoSet ELISA Development kits following the manufacturer's instructions (R&D Systems, Minneapolis, MN). The final levels were determined by detection with a luminescence counter (Packard Instrument Company Downers Grove, Illinois). All samples were thawed only once and assayed in triplicate.

### Statistical analysis

Statistical analyses were performed using SPSS statistical package, version 19.0 (SPSS, Inc., Chicago, IL). Analysis of variance (ANOVA) using Bonferroni adjustments (for age, WBC count, albumin level, CRP level, CEA level, IL‐1*β* level, IL‐6 level, and TNF*α* level) or Pearson chi‐square (χ^2^) test (for gender, location, and histologic differentiation) were used for multiple comparisons of different stages (Table 1). The Kaplan–Meier method was used to analyze survival, and the log‐rank test was used to examine any differences in survival. Univariate and multivariate analyses were used to study the association among variables (age, gender, tumor location, TNM stage, histologic differentiation, levels of albumin, CRP, CEA, IL‐1*β*, IL‐6, and TNF*α*, and survival). The 3‐year progression‐free survival rate (PFSR) was calculated using the Pearson chi‐square (χ^2^) test or Fisher exact test for an expected number per cell of <5. Multivariate analysis was performed using Cox's proportional hazards model. Differences were considered significant when the *P* value was <0.05.

**Table 1:** Clinicopathologic data for 164 CRC patients according to tumor stages

Variables expressed as number or mean ± SD	ALL	Stage I	Stage II	Stage III	Stage IV	*P* value^a^
Patient number	164	37	45	56	26	
Gender (male : female)	108:56	30:7	27:18	36:20	15:11	0.10
Age (median)	64.9 ± 13.7 (67.0)	62.7 ± 10.5 (59.5)	67.6 ± 15.8 (76.0)	64.1 ± 13.9 (65.0)	65.0 ± 13.5 (67.0)	0.42
Location (colon : rectum)	109:55	29:8	34:11	32:24	14:12	0.05
Histologic differentiations (well : moderate : poor)	50 : 104 : 10	19 : 18 : 0	14 : 29 : 2	12 : 39 : 5	5 : 18 : 3	0.02^a^
WBC (x 10^3^cells/*μ*L)	10.5 ± 4.4	10.8 ± 4.4	10.0 ± 3.9	9.9 ± 3.9	12.3 ± 5.4	0.11
Albumin (g/dL)	3.6 ± 0.7	3.9 ± 0.4	3.5 ± 0.8	3.7 ± 0.6	3.2 ± 0.7	<0.001^a^
CRP (mg/L)	24.6 ± 48.0	4.9 ± 6.5	27.8 ± 42.5	21.6 ± 52.6	51.7 ± 64.2	0.01^a^
CEA (ng/mL)	52.2 ± 525.2	2.3 ± 2.1	6.0 ± 9.8	7.9 ± 10.9	298.4 ± 1,312.5	0.08
IL‐1*β* (pg/mL)	17.7 ± 30.1	13.0 ± 4.1	22.1 ± 48.2	18.7 ± 27.6	14.6 ± 5.0	0.532
IL‐6 (pg/mL)	14.4 ± 54.8	6.8 ± 3.5	13.3 ± 38.8	18.5 ± 82.6	18.3 ± 41.9	0.763
TNF*α* (pg/mL)	68.7 ± 76.8	64.8 ± 14.6	96.7 ± 137.7	67.5 ± 40.3	60.3 ± 16.6	0.263
Proinflammatory cytokine intensity (0:1:2:3)	10:31:92:31	4:8:20:5	1:8:28:8	5:12:29:10	0:3:15:8	0.439
3‐year PFSR (%)	76.2	100	93.3	73.2	19.2	<0.001^a^

Proinflammatory cytokine intensity defined as the number of proinflammatory cytokine levels higher than median level (IL‐1*β* ≥ 10 pg/mL; IL‐6 ≥ 10 pg/mL; and TNF*α* ≥55 pg/mL).

CEA, carcinoembryonic antigen; CRC, colorectal cancer; CRP, C‐reactive protein; WBC, white blood cell, PFSR, progression‐free survival rate.

a *P* value was determined by ANOVA using Bonferroni adjustments (for age, WBC, albumin, CRP, CEA, IL‐1*β*, IL‐6 and TNF*α*) or chi‐square test (for gender, cancer location, histologic differentiation and proinflammatory cytokine intensity) for multiple comparisons.

## Results

The clinicopathologic characteristics of the 164 CRC patients enrolled are summarized in Table 1. The patients’ ages ranged from 18 to 94 years (average age 64.9 years). There were 108 men and 56 women. The tumor was located in the colon of 109 cases (66.5%) and in the rectum of 55 cases (33.5%). Using the criteria of 7th edition American Joint Committee on Cancer Staging System (AJCC), 37 patients (22.6%) had stage I, 45 patients (27.4%) had stage II, 56 patients (34.1%) had stage III, and 26 patients (15.9%) had stage IV disease. All patients had adenocarcinoma histology at the time of diagnosis. The histologic grade was assessed by World Health Organization criteria: 50 tumors (30.5%) were well differentiated, 104 tumors (63.4%) were moderately differentiated, and 10 tumors (6.1%) were poorly differentiated and/or undifferentiated. When patients were stratified by TNM stage, advanced stage was associated with poorer histologic differentiation, higher CRP levels, lower albumin levels, and inferior PFSR (Table 1). Advanced‐stage patients also showed a trend for higher CEA levels and more tumors in the rectum (0.05 < *P *<* *0.1). Tumor stage was not significantly associated with gender, age, WBC count, and levels of IL‐1*β*, IL‐6, and TNF*α* (Table 1).

**Figure 1:**
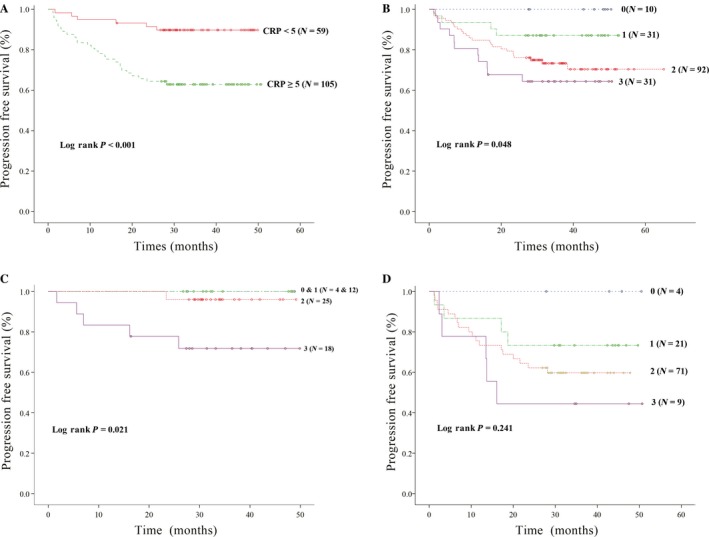
Progression‐free survival analysis for the 164 CRC patients stratified by (A) CRP level or (B) Proinflammatory cytokine intensity. (C) Progression‐free survival analysis for patients with CRP levels <5 mg/L stratified by proinflammatory cytokine intensity. (D) Progression‐free survival analysis for patients with CRP levels ≥5 mg/L stratified by proinflammatory cytokine intensity. 0 indicates no proinflammatory cytokines higher than the median; 1 denotes one proinflammatory cytokine higher than the median; 2 means two proinflammatory cytokines higher than the median; 3 represents all three proinflammatory cytokine levels higher than the median level.

We used serum levels of CRP and three proinflammatory cytokines, IL‐1*β*, IL‐6, and TNF*α*, to assess the relationship between inflammation status and the clinicopathologic features of CRC patients. Because the normal limit of CRP ranges from 0 to 5 mg/L in our institution and a study of hepatocellular carcinoma applied 5 mg/L as a threshold for the best sensitivity, specificity, and diagnostic accuracy 14, we used 5 mg/L as the cut‐off value for CRP in this study. There was no consensus of optimal cut‐off levels for all cytokines since an inherent enrollment difference exists in variations of disease, ethnicity, and measurement methodology among studies. We thus arbitrarily stratified patients according to the median level of each cytokine as a cut‐off value for analysis in this study (the median level was 10 pg/mL for IL‐1*β*, 10 pg/mL for IL‐6, and 55 pg/mL for TNF*α*). CRP >5 mg/L was associated with advanced tumor stage, lower albumin level, and lower PFSR (Table 2 and Fig. 1A). Patients with IL‐1*β *≥10 pg/mL or TNF*α *≥55 pg/mL had a lower albumin level and lower PFSR (Table 2). There were higher CRP levels in patients with IL‐1*β* levels ≥ 10 pg/mL and patients with TNF*α* ≥55 pg/mL were older (Table 2). Despite a trend for progression‐free survival benefit in patients with IL‐6 < 10 pg/mL (*P *=* *0.089), we could not detect any difference in clinicopathologic variables between patients with IL‐6 ≥ 10 pg/mL and IL‐6 < 10 pg/mL.

**Table 2:** The association between clinicopathologic features and proinflammatory cytokines and CRP for 164 CRC patients

Variables expressed as number or mean ± SD	IL‐1*β*<10 pg/mL	IL‐1*β* >10 pg/mL	*P* value^a^	IL‐6<10 pg/mL	IL‐6>10 pg/mL	*P* value^a^	TNF*α*<55 pg/mL	TNF*α* >55 pg/mL	*P* value^a^	CRP<5 mg/mL	CRP>5 mg/mL	*P* value^a^
Patient number	33	131		122	42		28	136		59	105	
Gender (male : female)	23:10	85:46	0.602	82:40	26:16	0.531	17:11	91:45	0.529	39:20	63:42	0.837
Age (median)	64.8 ± 13.465	64.9 ± 13.867	0.791	64.8 ± 13.866	65.2 ± 13.467	0.856	60.0 ± 10.660	65.9 ± 14.168	0.039^a^	64.63 ± 11.21165	65.75 ± 15.20168	0.625
Location (colon : rectum)	20:13	89:42	0.425	77:45	32:10	0.122	19:9	90:46	0.864	41:18	65:40	0.771
TNM stage												
I : II : III : IV	9:8:14:2	28:37:42:24	0.274	29:33:44:16	8:12:12:10	0.376	10:6:10:2	27:39:46:24	0.197	22:13:20:4	15:30:34:18	<0.001^a^
Histologic differentiations												
Well : moderate : poor	9:22:2	41:82:8	0.901	39:77:6	11:27:4	0.488	10:16:2	40:88:8	0.751	18:37:4	32:60:13	0.956
WBC (×10^3^cells/*μ*L)	10.1 ± 4,128	10.6 ± 4.5	0.569	10.7 ± 4.4	9.9 ± 4.3	0.272	11.8 ± 4.1	10.3 ± 4.4	0.093	10.8 ± 4.5	10.9 ± 4.2	0.909
Albumin (g/dL)	3.8 ± 0.5	3.5 ± 0.7	0.015^a^	3.6 ± 0.7	3.5 ± 0.7	0.415	4.0 ± 0.6	3.5 ± 0.7	0.002^a^	3.9 ± 0.5	3.3 ± 0.8	<0.001^a^
CRP (mg/dL)	12.7 ± 17.0	27.2 ± 52.2	0.019^a^	23.8 ± 43.0	26.9 ± 61.0	0.748	16.7 ± 37.3	26.4 ± 50.2	0.365	2.0 ± 1.4	42.8 ± 58.6	<0.001^a^
CEA (ng/mL)	7.0 ± 9.	63.5 ± 587.5	0.582	138.0 ± 889.1	170.3 ± 1036.8	0.327	7.4 ± 14.6	61.8 ± 578.7	0.614	7.9 ± 15.5	106.8 ± 786.5	0.336
IL‐1*β* (pg/mL)	8.4 ± 1.5	20.1 ± 33.2	0.046^a^	14.5 ± 6.6	27.1 ± 57.8	0.164	10.9 ± 3.6	19.1 ± 32.8	0.190	23.9 ± 49.2	14.7 ± 6.25	0.157
IL‐6 (pg/mL)	12.7 ± 35.3	14.8 ± 58.8	0.842	5.1 ± 2.4	41.6 ± 104.4	0.029^a^	6.7 ± 3.7	16.0 ± 60.0	0.415	12.8 ± 33.9	7.8 ± 11.0	0.236
TNF*α* (pg/mL)	59.6 ± 8.9	83.5 ± 85.2	0.111	71.1 ± 25.1	101.0 ± 144.6	0.190	50.7 ± 3.8	84.5 ± 83.2	0.034^a^	87.1 ± 123.8	76.0 ± 28.5	0.458
3‐year PFSR (%)	87.9	72.5	0.048^a^	78.7	66.7	0.089	92.9	72.1	0.020^a^	91.5%	65.8%	**<0.001** a

ALB, albumin; CEA, carcinoembryonic antigen; CRC, colorectal cancer; CRP, C‐reactive protein; TNM, tumor‐node metastasis; WBC, white blood cell, PFSR, progression‐free survival rate

a *P* value was determined by independent Student's *t*‐test (for age, WBC, albumin, CRP, and CEA) or chi‐square test (for gender, stage, location, histologic differentiation, and 3‐year PFSR) for different inflammatory cytokines criteria.

Although serum CRP level is under the regulation of proinflammatory cytokines 1, there was no strong correlation between CRP level and IL‐1*β* (*P *=* *0.433), IL‐6 ≥ 10 pg/mL (*P *=* *0.748) and TNF*α* ≥ 55 pg/mL (*P *=* *087), respectively in this study. We defined proinflammatory cytokine intensity of patients as the number of patients with proinflammatory cytokine levels higher than median level (IL‐1*β* ≥ 10 pg/mL; IL‐6 ≥ 10 pg/mL; and TNF*α* ≥ 55 pg/mL), and found a significant correlation between cytokine intensity and CRP level (*P *=* *0.048). In addition, we attempted to evaluate the effect of proinflammatory cytokines and other clinicopathologic parameters on PFSR. Although the univariate analysis showed that inferior PFSR was associated with a rectal tumor location (*P *=* *0.031), advanced tumor stage (*P *<* *0.001), serum albumin level <3.5 g/dL (*P *=* *0.021), CEA ≥ 5 ng/mL (*P *<* *0.001), CRP ≥ 5 mg/L (*P *<* *0.001), IL‐1*β* ≥ 10 pg/mL (*P *=* *0.048), TNF*α* ≥ 55 pg/mL (*P *=* *0.022) and higher cytokine intensity (Fig. 1B, *P* = 0.048), the multivariate analysis found tumor stage to be the only independent factor for PFSR (*P *<* *0.001).

Under different inflammation status shown in Table 3, the univariate analysis showed PFSR of patients with CRP <5 were significantly correlated with advanced stage (*P *<* *0.001), higher CEA level (*P *=* *0.017) and cytokine intensity (Fig. 1C, *P* = 0.021). Furthermore, the multivariate analysis revealed that in addition to tumor stage, cytokine intensity independently predicts PFSR of patients with CRP <5. Cytokine intensity was not correlated with PFSR (Fig. 1D, *P* = 0.241) but tumor stage was the only independent predictor for 3‐year PFSR in patients with CRP ≥ 5 mg/L in both univariate and multivariate analyses (Table 3).

**Table 3:** Statistical analysis of clinicopathologic features for PFS according to CRP level

	CRP <5 mg/L				CRP ≥5 mg/L			
	Univariate analysis		Multivariate analysis		Univariate analysis		Multivariate analysis	
	HR (95% CI)	*P* value	HR (95% CI)	*P* value	HR (95% CI)	*P* value	HR (95% CI)	*P* value
Age >65 year	4.472 (0.522–38.283)	0.172			1.069 (0.469–2.304)	0.864		
Sex (female vs. male)	0.983 (0.180–5.367)	0.984			0.758 (0.351–1.634)	0.758		
Location (Rectum vs. Colon)	1.151 (0.211–6.287)	0.871			2.273 (1.067–4.844)	0.033		
TNM Stage	16.153 (3.059–85.289)	<0.001	15.512 (2.135–112.731)	0.007	3.872 (2.218–6.759)	<0.001	2.504 (1.305–4.806)	0.006
Histologic differentiations	2.389 (0.556–10.265)	0.242			1.117 (0.564–2.216)	0.571		
WBC > 10,000 cells/mm³	1.964 (0.360–10.724)	0.436			1.904 (0.871–4.161)	0.107		
Albumin > 3.5 g/dL	0.889 (0.104–7.615)	0.915			0.768 (0.352–1.672)	0.768		
CEA > 5 ng/mL	8.002 (1.461–43.813)	0.017			3.498 (1.476–8.286)	0.004		
TNF*α* > 55 pg/mL	32.804 (0.150–768.440)	0.131			2.241 (0.531–9.465)	0.272		
IL‐1*β* > 10 pg/mL	26.308 (0.020–335.667)	0.154			1.784 (0.616–5.162)	0.286		
IL‐6 > 10 pg/mL	9.599 (0.980–82.266)	0.074			2.547 (1.112–5.838)	0.027		
Pro‐inflammatory cytokine intensity (0–3)	9.198 (1.214–69.699)	0.021	11.110 (1.165–105.971)	0.036	1.569 (0.680–3.621)	0.241		

PFS, progression‐free survival.

## Discussion

Disease severity and progression of CRC could be the sequelae of ongoing systemic inflammation determined by an increase in the serum CRP level, a well‐established standard measurement of inflammation. This link is supported by the following observations. First, CRC patients had a higher CRP level at the time of diagnosis than healthy individuals, and heightened CRP levels increase the risk of CRC occurrence 8, 15. Furthermore, proinflammatory cytokines regulate CRP expression and contribute to the inflammation‐induced tumorigenesis of CRC 1. IL‐1*β* initiates proinflammatory cascades and facilitates tumor spread 16. TNF*α* signaling activates transcription factor NFκB, thus promoting the expression of downstream inflammatory mediators that are involved in aberrant cell differentiation and proliferation 1. Clinically, higher IL‐1*β* expression was found in adenocarcinoma tissue relative to normal colon tissue 17. An increase in the TNF*α* level also increased the risk of CRC 18 and a decrease in the IL‐1*β* level decreased the risk of advanced CRC 19. IL‐6, an essential inflammation mediator downstream of the TNF*α* NFκB signaling pathway has been shown to correlate with CRC risk. Higher IL‐6 levels were associated with larger tumor size, advanced tumor stage, increased tumor occurrence, and decreased survival of CRC patients 20. Previously, Hamilton et al. measured 42 serum inflammatory mediators and reported that elevated CRP level was associated with inflammatory status and is an important prognostic indicator for CRC patients with liver metastasis 21. Finally, Sharma et al. analyzed 52 stage IV CRC patients according to the Glasgow Prognostic Score (GPS) and a panel of serum cytokines and chemokines, and found that patients with a high GPS score (both serum CRP level ≥10 mg/L and albumin level ≤3.5 g/dL) showed higher treatment toxicity and inferior prognosis. Serum IL‐6 and glycoprotein 130 levels were also associated with GPS 22. Taken together, this study found that elevated CRP level is correlated with high cytokine intensity that may affect survival in CRC patients and support the notion that the inflammation process mediated by a panel of proinflammatory cytokines indeed contributes to CRC development and prognosis.

The salient feature presented in this study was that cytokine intensity significantly predicts the progression of CRC patients with low‐level serum C‐reactive protein (CRP <5), but not high‐level (CRP ≥5), and deserves further discussion. To our knowledge, it is unknown what indices best reflect inflammation status associated with the prognosis of CRC patients. In addition to CRP level, inflammation indices such as prognostic nutrition index, neutrophil/lymphocyte ratio and platelet/lymphocyte ratio have been reported as prognostic factors for CRC 23, 24, 25. The results of this study suggest some patients were in high‐level inflammatory status but the serum level CRP level was relative low. In this circumstance, cytokine intensity shown this study may be able to reflect this high inflammation status and correlate with disease progression. We should pay more attention to patients with low CRP level and the proinflammatory cytokine intensity could be an alternative representative for inflammation state of CRC patients. Besides, many confounding variables such as smoking, obesity, dietary fatty acid intake, and socioeconomic status related to inflammation state 8 were not included into the analysis of this study. These factors may lead to design and survival bias as well as the status of varied inflammation despite the similar serum CRP levels detected. Finally, variants in the CRP gene, promoter polymorphisms of CRP, and proinflammatory cytokine levels modulated basal expression of serum CRP 26, 27. For example, three SNPs were associated with variation in serum CRP levels, CRP being increased in TT (rs1120864) and GG (rs2794521) genotypes and decreased in the AA genotype of rs1205 27.

As treatment options become more standardized, it becomes increasingly important to develop tools to select patients with high risk for CRC recurrence. The pro‐inflammatory cytokine intensity could provide valuable information to offer appropriate medical attention to CRC patients with low CRP level (CRP <5 mg/L) following standard treatment.

## Conflict of Interest

We declare no conflict of interest in terms of employment (other than primary affiliations), commercial grants, other commercial research support, ownership interest, membership to the consultant/advisory board, and honoraria from the speakers’ bureau.
